# Topics of the Month

**Published:** 1897-12

**Authors:** 


### TOPICS OF THE MONTH.

The courtesies of journalism are always pleasant to receive as well as to bestow. We take especial pleasure in reproducing the following editorial from the *Medical Record* of November 20, 1897:

### WHAT THE "MEDICAL RECORD" DOES.

The Buffalo Medical Journal, in an editorial in its issue of October last, referring to the late Moscow congress, says: "The *Medical Record* has published a full cabled synopsis of the proceedings, which is a marked instance of journalistic enterprise as well as lavish expenditure."

So far as we know this is the only reference which has been made in any American medical journal to what has been the most difficult and expensive attempt on our part to supply our readers with the news of the day, *pari passu* with its occurrence.

Remarkable as is the occasion, it is perhaps not to be wondered at, since any such reference would only go to show the lack of the same enterprise and expenditure on the part of any metropolitan journal so noticing it. We refer to it here simply to impress upon our readers the fact that the *Medical Record* never spares expense in providing material and news which the wide-awake members of the medical profession should have to keep themselves in the front rank of scientific progress. Its corps of regular correspondents all over the world is, unquestionably, we presume, the largest and most complete of any. We think it will hardly be considered egotism for us to say that it is the one journal which is indispensable to all who desire to be informed promptly of the progress of medical science and of medical news. While we make this claim for the *Medical Record*, we must reiterate what we have repeatedly said in these columns before—that no medical man who can afford it should neglect also to subscribe for and do what he can to support and encourage his local medical journal. With that and the *Medical Record* coming regularly to his table, he could be no better equipped if he subscribed to a dozen other journals, so far as periodical literature pertaining to his profession is concerned.

---

THE vending of spectacles will not be permitted on the streets of Buffalo. Mayor Jewett has invariably declined to grant licenses for such purposes. Moreover, in every license granted by the mayor the following prohibitive clause is added: "No eye-glasses are to be sold under this license." It has been stated in the newspapers that a lawyer proposes to test the matter in the courts, by applying for a writ of mandamus to compel the mayor to issue a license permitting the sale of spectacles. The mayor holds that he has power to refuse such a license, that he has habitually refused them because the sale of spectacles not scientifically tested before purchase might work great injury upon the eyes of people who buy them, and that he will willingly defend a suit to compel him to issue licenses in such cases. The attitude of the mayor is undoubtedly correct and deserves the approbation of all intelligent people.

---

A WASHINGTON dispatch to the *Tribune*, dated November 15, 1897, stated that

Surgeon-General Sternberg, of the Army; Dr. Horlbeck, of Charleston; Dr. Josiah Hartzell, of Canton, Ohio; Dr. Samuel H. Durgan, of Boston; Dr. A. H. Doty, of New York, and Dr. S. R. Olliphant, of New Orleans, the president of the Louisiana State Board of Health, all members of the American Public Health Association, called at the White House today. They saw the President and urged him to incorporate in his message a recommendation that a commission be appointed to go to Havana to study the subject of yellow fever and the manner in which it is brought to the United States. They declared that good regulations in Havana would do more to prevent yellow fever in the United States than the best quarantine regulations that could be adopted and enforced. The President said he would give their suggestions due consideration.

THE illegal practice of dentistry will not be permitted in the city of New York. The *Tribune* of November 17th said :

Gaetano Amato, the agent of the State Dental Society, yesterday caused the arrest of Claudius Adams, an employe in the Boston Dental Parlors, No. 44 East Fourteenth Street, on the charge of practising dentistry in this county without being licensed or registered, as the dental law requires. Adams, when taken before Magistrate Wentworth, in Jefferson Market Police Court, said that he was not guilty of the charge, on the ground that he did only mechanical work, but his position was not borne out by the evidence presented to the court, and he was held for trial.

Mr. Amato also caused the arrest of Ramon Lince, a Spaniard, who came recently from Medellin, South America, for violating the dental law, and arraigned him before Magistrate Flammer, in the Fourth District Court. Lince was practising dentistry at the Savoy Dental Parlors, No. 980 Third avenue. Lince was held under $200 bail for trial in Special Session.

---

THE United States Sanitary Commissioner at Constantinople, Dr. Spiridion C. Zavitziano, reports through the American Legation to the Surgeon-General of the Marine Hospital Service that, according to the official sanitary reports received from the different provinces of the Turkish Empire, there exists at present an epidemic of dengue fever in Adalia, on the Mediterranean coast of Asia Minor. In the other provinces public health is rather good. Only from Aleppo is announced an epizootic among oxen. He says :

"In Constantinople there exists always many cases of typhoid fever and some of smallpox, as well as other zymotic diseases. According to the statistics issued by the Bureau de la Mortalité of the Sanitary Board, there have been registered for the week ended September 28th, or, according to the new style, the 10th inst., two hundred deaths. I am glad to state that at the last sitting of the International Sanitary Commission the question about the way of building the hospitals in the Lazaretto of Camaran, whether they were to be built with or without ceilings, has been settled, and the necessary orders have been given to have them built with ceilings.

In conclusion Dr. Zavitziano writes: "The inhabitants of Feriklay, a suburb near Pera, where the cemetery of the Roman catholics is situated, have made severe complaints against the existence of the cemetery in the center of that suburb. Lately the cemetery has been enlarged. The International Sanitary Commission, to which these complaints have been addressed, has received from the French embassy a paper in which it is assured that the Roman catholic cemetery is managed in such a manner that public health will never be hurt. It is said, for instance, that the cemetery is divided into four squares. In the center are the common graves, in which the corpse must be left, according to the regulations, five years, but the graves are not used for other corpses until nine years elapse. In the surrounding line of the above-mentioned square the graves are left for five years, but can be kept for an indefinite time, provided a small fee is paid. Outside of these graves are those belonging forever to the families who bury there. These four squares are separated from each other by drain pipes going to two basins, which have the waste pipes by which the overflow goes to the main sewers. In time of epidemic the director of the cemetery buys unslacked lime in order to spread on the graves."

---

Dr. George Ben Johnston, president of the Southern Surgical and Gynecological Association, took for the subject of his presidential address at the St. Louis meeting The Prevalence of Specialism and who Should be Specialists. He divided specialists into two classes, the true and pseudo. The profession recognised the value of one, while the other derived his standing from the magic term "specialist," which was so ill-understood by the public. The real value of the specialist depended upon his broad cultivation in the department of general medicine. He would be all the better if he had a mind stored with facts, an experience derived from contact with all forms of disease and a judgment matured by observation and understanding. In fact, he said, the professional qualities of the true specialist might be likened to a massive pyramid, the finished capstone of which constituted the specialty. In the case of the pseudo specialist, this pyramid of learning was inverted, and having no base of general knowledge, it was top-heavy and unstable. The public had not been properly educated in the matter of specialism and was too much inclined to place credence in the claims of any one who called himself a specialist. The cause of this, Dr. Johnston said, was largely the fault of the profession which referred its most difficult cases to specialists, and from this the public drew the inference that all specialists were better than general practitioners. It could not discriminate between the true and the false. It was the duty of colleges and societies to institute reform in this line. Special societies should fix the standard and require that all applicants should conform to them and colleges should also take the matter in hand, sure of the support of the public and the general profession.

